# Peripheral primitive neuroectodermal tumor of the urinary bladder in an Arab woman with history of squamous cell carcinoma: a case report

**DOI:** 10.1186/1752-1947-3-6840

**Published:** 2009-04-29

**Authors:** Mohd Khaled Al Meshaan, Marwan Nayef, Talal Kwaider, Wolfgang Otto, Ken C Katchy

**Affiliations:** 1 Department of Urology, Sabah Hospital, Kuwait P.O. Box 3360, Safat 14013, State of Kuwait; 2 Department of Urology of Regensburg University, St. Josef's Hospital, Landshuterstraße 65, 93053 Regensburg, Germany; 3 Department of Pathology, Sabah Hospital, Kuwait P.O. Box 3360, Safat 14013, State of Kuwait

## Abstract

**Introduction:**

Peripheral primitive neuroectodermal tumors of the urinary bladder are rare and tend to occur in an older age group than do their counterparts in bones and soft tissue.

**Case presentation:**

We report a case of peripheral primitive neuroectodermal tumor of the urinary bladder in a 67-year-old woman of Arab origin. She had undergone transurethral resection followed by chemotherapy because of pulmonary metastasized muscle-invasive squamous cell carcinoma of the bladder in 2005. One year later, she first presented with a history of repeated hematuria in our institution. Performing cystoscopy any tumor could be detected. Control cystoscopy two months later showed a tumor mass of 3 cm in diameter at another location than described for the first tumor. After perforating by transurethral resection partial bladder resection had to be done. Tissue specimen after pathological analysis revealed a peripheral primitive neuroectodermal tumor with tumor cells reactive to cluster of differentiation 99, neuron-specific enolase and S100 protein and stained negative for other markers such as cytokeratins, epithelial membrane antigen, desmin, smooth muscle actin, chromogranin and leucocyte common antigen. Staging computerized tomography was especially free from any hint on organ metastasis, but the patient died due to a cardiac problem only a few months later.

**Conclusions:**

To the best of our knowledge, we report the eighth case of bladder peripheral primitive neuroectodermal tumors in literature and the first concerning an Arab patient. It is also the first presentation of a peripheral primitive neuroectodermal tumor patient with a history of squamous cell carcinoma of the bladder. As in other cases, expression of single-chain-type 1 glycoprotein and neural markers was positive and the disease was at an advanced stage at the time of diagnosis.

## Introduction

Peripheral primitive neuroectodermal tumors (pPNET) are malignant small round-cell tumors that occur predominantly in bones and soft tissue of children and young adults [1], only in rare cases in other organs and in older patients [2]. pPNET belong to the Ewing's sarcoma family of tumors and share the same immunohistochemical profiles and molecular genotypes [3]. Tumor cell staining of these masses was shown to be positive for MIC2 gene product (CD99) and neural markers such as neuron-specific enolase (NSE), vimentin, S100 protein or synaptophysin [3]-[6]. Primitive neuroectodermal tumors of the urinary tract are very rare, more often occurring in renal masses than as bladder tumors [3]. To our knowledge, only 7 cases of primary pPNET of the urinary bladder have been reported so far [3]-[9], often concerning much older patients than those presenting with neuroectodermal tumors of other organs [5]-[7]. Poor prognosis necessitates multimodal treatment with chemotherapy and radiotherapy, following surgical measures [3].

## Case presentation

We report the first case of a peripheral primitive neuroectodermal tumor of the urinary bladder in an Arab country. The Department of Surgery of Al Sabah Hospital, Kuwait admitted a 67-year-old diabetic and hypertensive Arab woman in February 2006, due to severe hematuria and fever. In January 2005, the woman had already been treated in a hospital of Great Britain for repeated hematuria for the duration of one year. During this initial admission, cystoscopy revealed an intrinsic urinary bladder mass that was resected. Histologically, it was reported as poorly differentiated squamous cell carcinoma (pT_2_G_3_). Computerized tomography (CT) scan showed diffuse but irregular thickening of the bladder wall, pelvic lymphadenopathy and multiple small bilateral pulmonary metastases. Mild hydronephrosis was noted in the right kidney, other abdominal organs were essentially normal. The patient received three courses chemotherapy, to which her response was excellent, and was discharged home in good condition. In November 2005, the patient underwent a follow-up cystoscopy that showed a recurrent malignant urinary bladder tumor which was completely resected. A CT scan of thorax, abdomen and pelvis, that did not show evidence of cancer progression, and a negative bone scan completed the follow-up.

**Figure 1:**
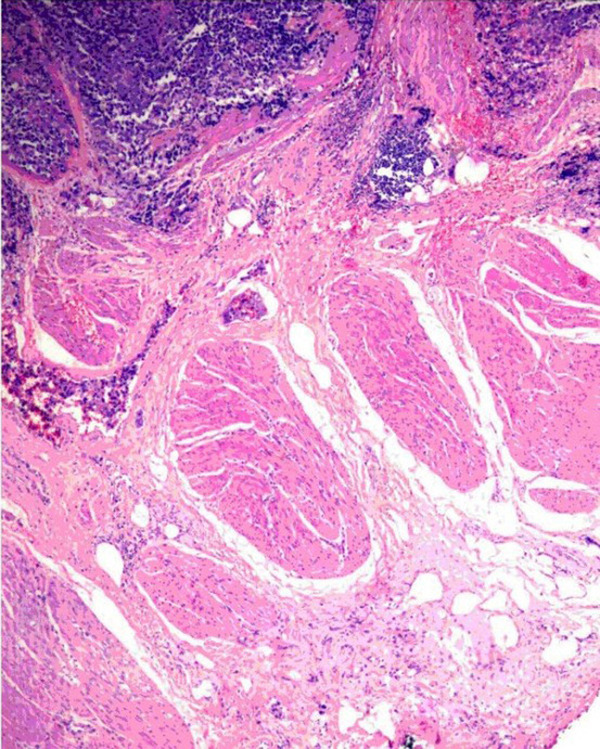
**Malignant small round-cells arranged in irregular masses and clusters**. Tumor is seen above the muscularis propria of the bladder wall in this field (HE, 40x).

**Figure 2:**
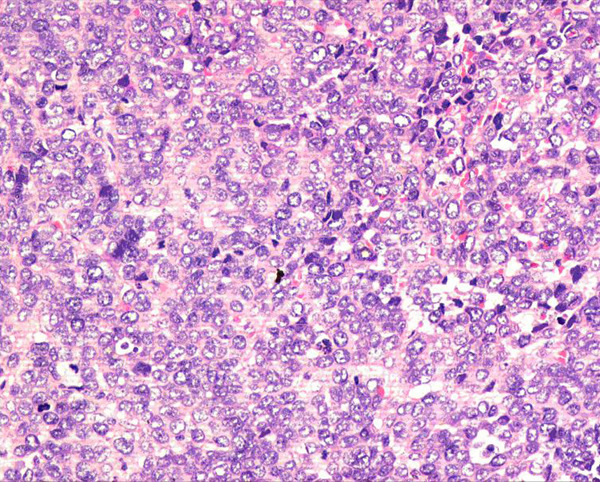
**Predominantly small cells with round vesicular nuclei arranged in diffuse sheet (HE, 200x)**.

**Figure 3:**
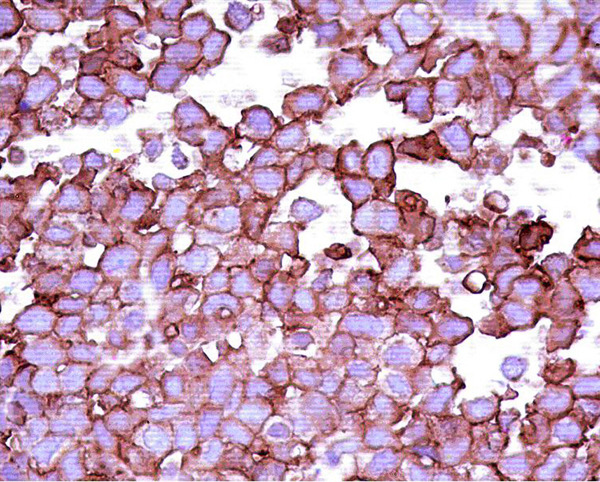
**The small round-cells of the pPNET in our case showed positivity for MIC2 gene (CD99+) (400x)**.

On first admission at our institution in February 2006, the patient was pale and pyretic. Her hemoglobin was 7.7 g/dl and the blood culture showed an E. coli infection. Ultrasonography revealed hydronephrosis on the right side and a thick bladder wall without apparent masses, verified by cystoscopy. Based on the sensitivity report, she was treated with ciprofloxacin and a 3-way irrigation catheter was inserted to rinse the bladder. Although her general condition improved, mild hematuria persisted. Follow-up cystoscopy in April 2006 showed a tumor located at the posterior wall and the dome of the urinary bladder (another site than described for the squamous cell carcinoma). As an initial attempt at transurethral resection (TUR) resulted in perforation, a partial bladder resection was performed. A post-surgery CT of the thorax, abdomen and pelvis showed mild dilatation of the right renal pelvis, a thickened urinary bladder wall without an exophytic mass and no evidence of lung metastasis. The tumor specimen gained during surgery consisted of grey-brown tissue measuring 3 × 2.5 × 1 cm. Pathohistology showed features of a malignant small round-cell tumor (Figures 1 and 2), with frequent mitosis, apoptosis, necrosis, vascular invasion and deep infiltration of the muscularis propria. The tumor cells expressed CD99 (MIC2 gene, Figure 3), NSE and S100 protein, but were non-reactive to cytokeratins, epithelial membrane antigen, desmin, smooth muscle actin, chromogranin and leucocyte common antigen. A diagnosis of peripheral primitive neuroectodermal tumor (pPNET) obviously due to secondary tumor development was made. The patient was discharged in good condition but died of cardiac-related problems in October 2006.

## Discussion and conclusions

Peripheral primitive neuroectodermal tumors (pPNET) of the urinary bladder are rare, aggressive masses that partly tend to occur at a much older age than do their bone and soft tissue counterparts in the Ewing's sarcoma family. It is said, that concerned patients are either of young age like in other small round-cell tumors [4,8,9] or of advanced age [5]-[7] as in our case. In this age group, these tumors may be confused with the more frequent transitional cell carcinomas. Besides, pPNET must be differentiated from other rare tumors of the urinary bladder, such as embryonal rhabdomyosarcoma that may also occur in older age groups [10]. However, pPNET following or preceeding squamous cell carcinoma as in our case presentation was never reported before. Immunohistochemistry and, where available, molecular biology, are considered necessary in the evaluation of undifferentiated or poorly differentiated bladder tumors. In the present case, the immunohistochemistry profile supported the diagnosis of pPNET. As in most reported cases of pPNET, our patient showed positivity for neuron-specific enolase and MIC2 gene [3,5,6,8]. Other neural markers showed different results in pPNET diagnostics. Whereas S100 protein was positive in our case, this marker showed different results in other pPNET patients with positive [3,4,6] and negative staining [8,9]. Cytokeratins, epithelial membrane antigen, desmin (CD33), smooth muscle actin, chromogranin and leucocyte common antigen (CD3 und CD20) were not significant markers in this case, in accordance with the results of Desai and Banerjee et al [8,9].

Patients in some cases already had metastases at the time of diagnosis and this suggested a poor prognosis [5]-[8], however, it must be noted that the outcome of pPNET of the urinary bladder cannot be predicted. On the one hand, this is due to its rarity and thus missing long-term data, and, on the other hand, because it often affects older people with concomitant diseases, which may render differentiation of cause of death difficult. While our patient died soon after discharge of cardiac-related problems after a symptomatic period of 30 months of bladder tumor disease (squamous cell carcinoma and pPNET) in total, disease-free survival durations of up to 3 years have been reported [4].

## List of abbreviations

CD: Cluster of differentiation; CT: Computerized tomography; MIC2: Single-chain type-1 glycoprotein; NSE: Neuron-specific enolase; pPNET: Peripheral primitive neuroectodermal tumor; TUR: Transurethral resection.

## Consent

Written informed consent was obtained from deceased patient relatives for publication of this case report and accompanying figures. A copy of the written consent is available for review by the Editor-in-Chief of this journal.

## Competing interests

The authors declare that they have no competing interests.

## Authors' contributions

MKA supervised treatment of the patient and drafted the manuscript, MN, TK and WO helped to draft the manuscript. KK performed the histopathological analysis and helped to draft the manuscript.
